# Specialized care for people with AIDS in the state of Ceara, Brazil

**DOI:** 10.1590/S0034-8910.2015049006028

**Published:** 2015-10-05

**Authors:** Nathália Lima Pedrosa, Vanessa da Frota Santos, Simone de Sousa Paiva, Marli Teresinha Gimeniz Galvão, Rosa Lívia Freitas de Almeida, Ligia Regina Franco Sansigolo Kerr

**Affiliations:** I Programa de Pós-Graduação em Enfermagem. Universidade Federal do Ceará. Fortaleza, CE, Brasil; II Prefeitura Municipal de Fortaleza. Fortaleza, CE, Brasil; III Departamento de Enfermagem. Faculdade de Farmácia, Odontologia e Enfermagem. Universidade Federal do Ceará. Fortaleza, CE, Brasil; IV Programa de Pós-Graduação em Saúde Coletiva. Universidade Federal do Ceará. Fortaleza, CE, Brasil; V Departamento de Saúde Comunitária. Faculdade de Medicina. Universidade Federal do Ceará. Fortaleza, CE, Brasil

**Keywords:** Acquired Immunodeficiency Syndrome, Ambulatory Care, Equity in the Resource Allocation, Health Vulnerability, Ecological Studies

## Abstract

**OBJECTIVE:**

To analyze if the distribution of specialized care services for HIV/AIDS is associated with AIDS rates.

**METHODS:**

Ecological study, for which the distribution of 10 specialized care services in the Ceara state, Northeastern Brazil, was obtained, and the mean rates of the disease were estimated per mesoregion. We evaluated 7,896 individuals who had been diagnosed with AIDS, were aged 13 years or older, lived in Ceara, and had been informed of their condition between 2001 and 2011. Maps were constructed to verify the relationship between the distribution of AIDS cases and institutionalized support networks in the 2001-2006 and 2007-2011 periods. BoxMap and LisaMap were used for data analysis. The Voronoi diagram was applied for the distribution of the studied services.

**RESULTS:**

Specialized care services concentrated in AIDS clusters in the metropolitan area. The *Noroeste Cearense* and west of the *Sertoes Cearenses *had high AIDS rates, but a low number of specialized care services over time. Two of these services were implemented where clusters of the disease exist in the second period. The application of the Voronoi diagram showed that the specialized care services located outside the metropolitan area covered a large territory. We identified one polygon that had no services.

**CONCLUSIONS:**

The scenario of AIDS cases spread away from major urban areas demands the creation of social support services in areas other than the capital and the metropolitan area of the state; this can reduce access barriers to these institutions. It is necessary to create specialized care services for HIV/AIDS in the *Noroeste Cearense* and north of Jaguaribe.

## INTRODUCTION

Social support requirements involve, in addition to that given by family and friends, social relationships at institutionalized facilities. Social support networks can provide direct and indirect services, which are part of or complementary to the health service. These services can improve quality of life and disease management and help the adoption of new lifestyles – actions that may culminate in autonomy for the individual and his/her consequent empowerment.^1^

The specialized care services (SCS) for people living with HIV/AIDS seek to promote material and emotional support, as well as provide care to the patient and their families, which can take the form of clarifying rights, prevention policies, and health promotion.^12^ ^,^ ^17^ The SCS are free services provided by the Brazilian Unified Health System (SUS). These services provide comprehensive, humanized, individual care, tend to users’ vulnerabilities with multidisciplinary teams, and identify health care challenges, aiming to achieve user satisfaction by meeting the patients’ needs and expectations.^2^ ^,^ ^15^

Health promotion policies involve intersectoral actions that are always directed towards improving the living conditions for the individual and the population. In the midst of the AIDS epidemic and using the SUS principles and guidelines as a basis, activities that focus on the population’s quality of life have emerged. Public policies geared towards these individuals were encouraged, ensuring the free distribution of antiretroviral therapy and looking to expand the SCS.^8^

In Brazil, the HIV/AIDS epidemic is characterized by the geographical diffusion of the disease towards small cities,^10^ which have little access to specialized health institutions and, consequently, a greater chance of late diagnosis and inadequate management of its associated diseases. In the Brazilian state of Ceara, 96.0% of all its cities have identified at least one case of AIDS.^a^

Despite it not being an isolated factor, access to health services has been considered an important, impacting factor on the use of health-related services. Thus, the location of health facilities is a critical variable for providing quality health care. Identifying deficiencies in care coverage and disadvantaged groups is essential for managing health resources.^20^

Despite the advances in AIDS research, there are holes regarding the study of distributing specialized care services, which limits a true picture of these patients access to such institutions. The existence of accessible institutions that can meet the requirements that result from AIDS, along with the current dynamics of the disease, is a necessary challenge for modern society. The aim of this study was to analyze whether the distribution of the Specialized Care Services in HIV/AIDS is associated with AIDS rates.

## METHODS

This ecological study used a geographical information system (GIS) as a tool. These types of systems make it possible to build a set of data and arrange them to process spatially referenced information using computational techniques, which enables the conditions of vulnerable populations to be evaluated in relation to one variable.^3^

The address and first year of operation of 10 SCS specialized in AIDS and included in the institutional support network for the disease in Ceara, Northeastern Brazil, were taken from the *Cadastro Nacional de Estabelecimentos de Saúde* (CNES – National Registry of Health Facilities),^b^ which provides information regarding the health care network in the three spheres of government (municipal, state and federal). Thus, it was possible to georeference the services on a map built to include AIDS rates.

Each city was an analysis unit. The variables studied were the mean AIDS rates by municipality and the geographic location of the SCS. We used the geographical coordinates of addresses of institutions, and the code from the Brazilian Institute of Geography and Statistics (IBGE) referring to the city of residence of people who reported having the disease within the studied period.

**Figure 1:**
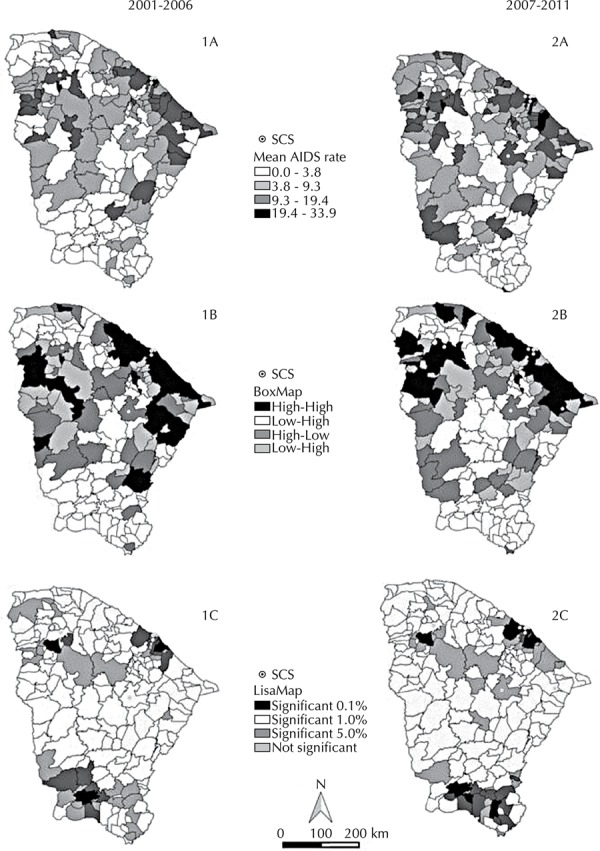
Map of the cities and mesoregions of the state of Ceara, according to the 2010 demographic census. Northeastern Brazil, 2014.

The state of Ceara is located in the Northeastern Region of Brazil. This state is divided into seven mesoregions, comprising 184 cities spread over an area of approximately 148,825.6 km^2^. In 2012, its population was estimated at approximately 8.6 million inhabitants, with a population density of 57.8 inhab./km^2^ (Figure 1).^a^

The evaluation included 7,896 individuals diagnosed with AIDS, aged 13 years or older, who lived in Ceara, and had been informed of their condition between 2001 and 2011, based on records from the *Sistema de Informação de Agravos de Notificação* (SINAN – Notifiable Diseases Information System).^c^ Duplicate cases and repeated notifications were excluded.

The mean rate of AIDS per city was calculated for the 2001-2006 and 2007-2011 periods. In the first period, the mean number of cases and the mean population were respectively obtained through the total number of cases between 2001 and 2006 and through the total added population in those six years, and both totals were then divided by six. In the second period (a five-year period), the mean number of cases and the mean population were obtained by dividing the totals by five. The AIDS rate in each period represents the mean number of cases divided by the mean population, multiplied by 100,000 inhabitants.

The global Moran’s index was also used, regarding the mean rate, to test for the existence of spatial autocorrelation. This index, whose calculation tests whether surrounding areas differ from what would be expected in a pattern of complete spatial randomness, gives values ranging from -1 to +1. Spatial aggregation is expressed for positive values, while inverse autocorrelation expresses negative values; spatial autocorrelation is absent when its value tends to zero.^16^

The presence of spatial aggregates was evaluated by the local Moran’s index (Local Indicators of Spatial Association – LISA) and can be seen on the LisaMap. This index makes it possible to identify spatial dependence and quantify the degree of spatial association at each location of the sample set. The values of significance are divided into four groups: non-significant, with a 95.0% significance, with a 99.0% significance, and with a 99.9% significance.^14^

The BoxMap was used to graphically visualize a Moran scatter plot, which divides the area into quadrants with specific characteristics. The areas located in quadrants Q1 (High-High) and Q2 (Low-Low) have positive spatial autocorrelation, meaning that these are cities with AIDS rates similar to their neighbors, characterizing spatial aggregates. Quadrants Q3 (High-Low) and Q4 (Low-High) have negative spatial autocorrelation, which represents cities that have disparate disease rates compared with nearby cities.^14^

To describe the areas under the influence of the SCS, a Voronoi diagram was built based on the points formed by the geographical coordinates of each institution’s address, distributed over the two study periods. This diagram makes it possible to define regions associated with each individual point on a map, rather than segmenting a geographical area into legally defined boundaries, whose polygons are divided in the space according to points, events, or both.^7^ This approach, which is useful in epidemiological research, enabled clusters of points to be detected in a given space and time.^13^

Maps were constructed for both periods, 2001 to 2006 and 2007 to 2011, along with the AIDS rates and the spatial distribution of the SCS. The state’s available health facilities were analyzed to find whether they were distributed near areas with high concentrations of AIDS cases. QuantumGis^®^ v.2.4.0 software was used.

This study is part of expanded research on the spatial distribution and social determinants of health in AIDS sufferers in the Brazilian state of Ceara, approved by the The Ethics Committee of the State Department of Health in Ceara (Protocol 203,911).

## RESULTS

**Figure 2:**
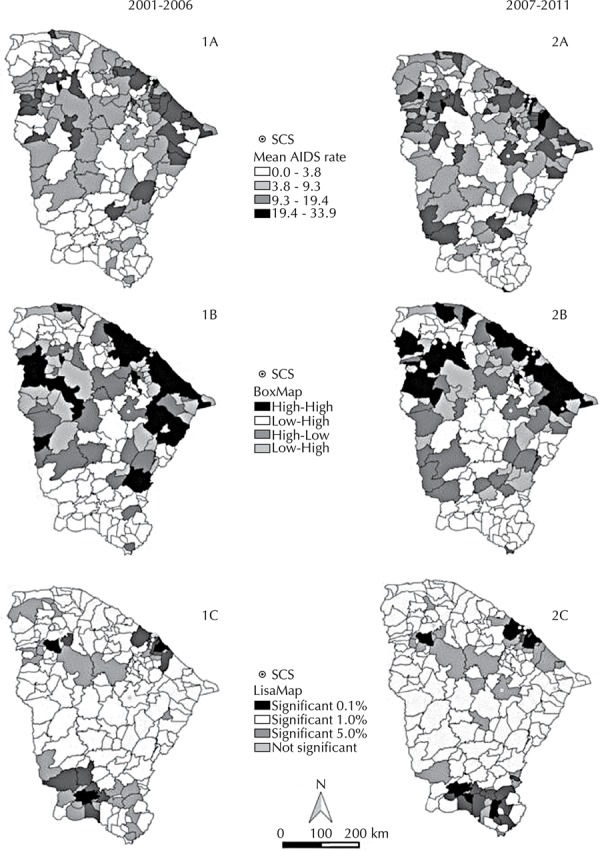
Geographical distribution of Specialized Care Services (SCS), mean rates (maps 1A and 2A), BoxMap (maps 1B and 2B) and LisaMap (maps 1C and 2C) of the AIDS rates according to the Moran scatter plot, in the state of Ceara, Northeastern Brazil, 2001-2011.

The distribution of AIDS throughout the two periods under study showed clusters of cases in the following regions: the capital of Ceara and its surroundings; *Noroeste Cearense*, next to the border with the state of Piaui; and north of the Jaguaribe city (Figure 2 - Maps 1A and 2A). Low concentrations were observed in the cities and neighboring areas of the central region and *Sul Cearense*.

Almost the entire Ceara coastline showed high rates of AIDS (Figure 2 - Maps 1A and 2A), and the relationship of cities neighboring the coastline had the same High-High pattern (BoxMap – Figure 2 - Maps 1B and 2B). We observed an expansion of the disease in the west of the state, upon comparing the first and the second period, with the highest amount of cities with the High-Low or Low-High standard. In turn, the LisaMap (Figure 2 – Maps 1C and 2C) shows that the AIDS rates and their spatial correlation with the neighboring regions in the 2001 to 2006 period are statistically significant in Fortaleza (state capital), as well as in part of the Metropolitan Region, in Sobral (central region of *Noroeste Cearense*) and in *Sul Cearense*. After the Lisa method was applied, significant growth was observed in cases of AIDS in the southwestern cities of the *Sertões Cearenses *(mesoregion in Ceara), which had previously not been affected by the disease. Between 2007 and 2011, there was a significant distribution of AIDS in as many cities as in the previous period, and still in the same regions (Metropolitan, *Noroeste Cearense* and *Sul Cearense*).

While dealing with the distribution of SCS along with the distribution of AIDS in Ceara (Figure 2), eight institutions were observed being initially concentrated in Fortaleza, showing a statistically significant cluster of AIDS cases. However, areas such as the *Sertões Cearense* and south of Jaguaribe had clusters of the disease, but without any SCS nearby. In the second period, two more SCS opened: one in Fortaleza and other north of Jaguaribe, which is a location with high rates of the disease. However, there was only one SCS in the *Noroeste Cearense*, a region with significant clustering of cases, with no other service having been opened.

**Figure 3:**
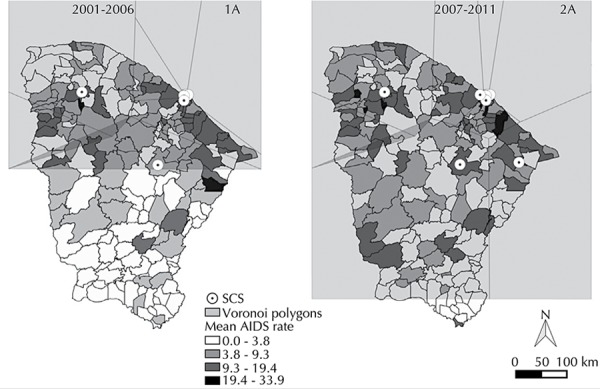
Distribution of Specialized Care Services (SCS) and Voronoi polygons. Ceara, Northeastern Brazil, 2001-2011.

The Voronoi diagram, showing the distribution of the SCS (Figure 3), made it possible to observe that the services further away from state capital covered a larger geographical area. Between 2001 and 2006 (Figure 3 - Map 1A), the polygons referring to the SCS did not manage to cover the entire state’s length, indicating possible locations that are a long way from the service. Over the 2007 to 2011 period (Figure 3 - Map 1A), the polygons covered almost all of Ceara. Furthermore, one polygon with no SCS was located in the western portion of the state.

## DISCUSSION

Despite the concentration of AIDS in the metropolitan area, the state of Ceara has clusters of the disease in other regions, such as north of Jaguaribe, *Noroeste Cearense *and west of the *Sertões Cearenses*, which were observed throughout the two periods under study. Regarding the spread of the epidemic away from major urban areas, access to health services impacts on the quality of life for people living with HIV/AIDS.^5^ Centralizing the specialized care services instead is confronted with the epidemic’s geographic expansion, which places obstacles in front of the population living farthest away from having access to care, leaving them with less attention and priority.^11^

The predominance of SCS and clusters of AIDS in the metropolitan region was also verified, which may influence the patients’ accessibility to these services. There is a consensus that concentrating care in reference units can make it difficult for the patients to geographically access it, which is due to the traveling costs, time off work, among other factors. However, people who exhibit stigmatizing diseases, such as HIV/AIDS, prefer their homes to be far away from these units, as there is a wish to maintain anonymity and not to be recognized by other individuals who use the service.^1^

Research indicates the disproportionate number of SCS in the northwest and west of the *Sertões Cearenses* in relation to AIDS rates, whose values are high. However, one SCS was created in Jaguaribe during the second period, which responded to the area’s demand regarding care to be given to individuals living with HIV/AIDS.

The spatial distribution of the health units directly has a direct effect on geographical accessibility that, in the case of large distances, combined with time and lack of transport, hampers people in need of specialized care from receiving it.^6^

Still, managers must decide to implement a health service based on demand. This demand can be monitored by various health information systems along with researchers, academic institutions, and users of the SUS,^19^ assisting in logistical planning and when looking to open new services. In Brazil, these systems are rarely used in smaller cities, which can lead to health services being implement in places of low demand, at the expense of areas that need more health units.^9^

The *Centro-Sul Cearense* did not have any SCS. The distance between the smaller cities and the major urban centers, where the SCS are concentrated, makes access to resources provided by these services difficult for many. As a result, people become more vulnerable to the disease’s consequences, a challenge for advances against this epidemic.^1^

This study is limited by the absence of similar research, making it difficult to challenge data obtained with this situation at other locations. It was not possible to find information on the minimum number of SCS recommended based on the population size or number of people living with HIV/AIDS, which makes wider analysis on service availability in the state difficult.

Studies that have a qualitative approach to understand the social and behavioral dynamics of the individual/SCS ratio is required, which could broaden the understanding of these distances or proximities with support services.

Further information regarding the AIDS epidemic is still required to make geoprocessing and spatial analysis feasible. In addition, it is necessary that the information generated through the research must reach health care institutions and policy makers so that the reality encapsulated by the studies can be changed.^17^

This study showed that the countryside regions of Ceara have areas with both high rates of AIDS and low numbers of SCS, and the reverse, regarding areas that were chosen to open new services, such as those areas that benefited during the second period under study. Basic health care can contribute for helping these patients, as accessibility to it is greater, which saves the secondary and tertiary institutions for more complex situations.

Methodological resources could be expanded, based on the idea that SCS interfere with the health-disease process of people living with HIV/AIDS, because they provide help during health care activities, as well as the prevention and treatment of these patients.

## Footnotes

a Instituto de Pesquisa e Estratégia Econômica do Ceará. Ceará em números. Fortaleza: Instituto de Pesquisa e Estratégia Econômica do Ceará; 2014 [cited 2014 May 21]. Available from: http://www.ipece.ce.gov.br/

b Ministério da Saúde. Cadastro Nacional de Estabelecimentos de Saúde. Brasil: Cadastro Nacional de Estabelecimentos de Saúde; 2015 [cited 2015 May 2]. Available from: http:// http://cnes.datasus.gov.br/

c DATASUS. Ministério da Saúde. Sistema de Informação de Agravos de Notificação. Brasil: Sistema de Informação de Agravos de Notificação; 2012 [cited 2012 May 2]. Available from: http://www2.datasus.gov.br/
