# Supplementary material for: Specialized care for people with AIDS in the state of Ceara, Brazil
Source: Rev Saude Publica. 2015 Oct 5;49:77. doi: 10.1590/S0034-8910.2015049006028 (PMC4603268; doi:10.1590/S0034-8910.2015049006028)
Supplement: Translation

# Translation: Assistência especializada para pessoas com aids no estado do Ceará, Brasil

**Authors:** Nathália Lima Pedrosa, Vanessa da Frota Santos, Simone de Sousa Paiva, Marli Teresinha Gimeniz Galvão, Rosa Lívia Freitas de Almeida, Ligia Regina Franco Sansigolo Kerr

**Affiliations:** I Programa de Pós-Graduação em Enfermagem. Universidade Federal do Ceará. Fortaleza, CE, Brasil; II Prefeitura Municipal de Fortaleza. Fortaleza, CE, Brasil; III Departamento de Enfermagem. Faculdade de Farmácia, Odontologia e Enfermagem. Universidade Federal do Ceará. Fortaleza, CE, Brasil; IV Programa de Pós-Graduação em Saúde Coletiva. Universidade Federal do Ceará. Fortaleza, CE, Brasil; V Departamento de Saúde Comunitária. Faculdade de Medicina. Universidade Federal do Ceará. Fortaleza, CE, Brasil

**Keywords:** Síndrome de Imunodeficiência Adquirida, Assistência Ambulatorial, Equidade na Alocação de Recursos, Vulnerabilidade em Saúde, Estudos Ecológicos

## Abstract

**OBJETIVO:**

Analisar se a distribuição dos serviços de assistência especializada em HIV/aids está associada às taxas de aids.

**MÉTODOS:**

Estudo ecológico, pelo qual foi obtida a distribuição de 10 serviços de assistência especializada em HIV/aids, no estado do Ceará. As taxas médias da doença foram calculadas por mesorregião. Estudaram-se 7.896 indivíduos notificados com aids, com idade igual ou superior a 13 anos, residentes no Ceará, no período de 2001 a 2011. Construíram-se mapas para verificar relação entre distribuição dos casos de aids e das redes institucionalizadas de apoio nos períodos de 2001 a 2006 e 2007 a 2011. Utilizou-se BoxMap e LisaMap para análise dos dados. Aplicou-se o diagrama de Voronoi para os serviços estudados.

**RESULTADOS:**

Verificou-se concentração dos serviços de assistência especializada nos *clusters* de aids na Região Metropolitana do estado. O Noroeste Cearense e o oeste dos Sertões Cearenses apresentaram elevadas taxas de aids, porém baixa quantidade de serviços especializados ao longo do tempo. Houve implantação de dois serviços especializados no segundo período onde existem aglomerados da doença. A aplicação do Diagrama de Voronoi mostrou que os serviços localizados fora da Região Metropolitana abrangem grande extensão territorial. Foi identificado polígono sem nenhum serviço.

**CONCLUSÕES:**

O cenário de interiorização dos casos de aids demanda a necessidade de criação de serviços de apoio social além da capital e da Região Metropolitana do estado, a fim de diminuir as barreiras de acesso a essas instituições. É necessário criar serviços de assistência especializada em HIV/aids no Noroeste Cearense e no norte do Jaguaribe.

## INTRODUÇÃO

As demandas de apoio social envolvem, além da família e amigos, relações sociais em meios institucionalizados. Redes sociais de apoio podem constituir-se da prestação de serviços diretos e indiretos, integrantes ou complementares ao serviço de saúde. Esses serviços melhoram a qualidade de vida e o gerenciamento de doenças e promove a adoção de novos estilos de vida, ações que podem culminar com a autonomia do indivíduo e o consequente empoderamento.^1^

O Serviço de Assistência Especializada (SAE) às pessoas vivendo com HIV/aids busca promover ações de apoio material e emocional, além de assistência à clientela e aos seus familiares, esclarecendo direitos, políticas de prevenção e promoção da saúde.^12^ ^,^ ^17^ Os SAE compõem um serviço gratuito oferecido pelo Sistema Único de Saúde (SUS). São espaços para atenção integral do indivíduo, com assistência humanizada, atuando nas suas vulnerabilidades por meio de equipe multidisciplinar e identificando desafios do cuidado, com o objetivo de obter satisfação do usuário por meio do atendimento das necessidades e expectativas dos pacientes.^2^ ^,^ ^15^

A política de promoção da saúde envolve ações intersetoriais sempre direcionadas para melhoria das condições de vida do indivíduo e da população. Em meio à epidemia da aids e tendo como base os princípios e diretrizes do SUS, emergem ações que visam à qualidade de vida dessa população. Houve incentivo às políticas públicas voltadas para essa clientela, garantindo a distribuição gratuita da terapia antirretroviral e a expansão dos SAE.^8^

No País, a epidemia de HIV/aids vem caracterizando-se pela interiorização dos casos, ou seja, difusão geográfica da doença e em direção a municípios de pequeno porte,^10^ locais onde o acesso às instituições especializadas de saúde é limitado, ampliando a chance de diagnóstico tardio e de manejo inadequado das doenças associadas. No Ceará, 96,0% de todos os municípios já identificaram pelo menos um caso de aids.^a^

Apesar de não ser fator isolado, o acesso aos serviços de saúde tem sido considerado fator importante, gerando impacto no uso dos serviços de saúde relacionados. Assim, a localização das instalações de saúde é fundamental para prestação de assistência de qualidade. Identificar deficiências na cobertura e populações desfavorecidas é essencial para a gestão dos recursos de saúde.^20^

Apesar dos avanços nas pesquisas sobre aids, existem lacunas no estudo da distribuição dos serviços de assistência especializada, limitando uma visão real sobre a acessibilidade dessa clientela às instituições. A existência de instituições acessíveis que atendam às necessidades consequentes da aids de acordo com a dinâmica atual da doença é um desafio necessário para a sociedade moderna. Este estudo objetivou analisar se a distribuição dos serviços de assistência especializada em HIV/aids está associada às taxas de aids.

## MÉTODOS

Neste estudo ecológico, empregou-se como ferramenta um Sistema de Informação Geográfica (SIG). Essa classe de sistemas permite constituir um conjunto de dados e organizá-los no tratamento de informações, espacialmente referenciadas, por meio de técnicas computacionais, possibilitando a avaliação das condições de populações vulneráveis em relação a uma variável.^3^

Os endereços e os anos de início de atendimento de 10 SAE vinculados à aids e que fazem parte da rede de apoio institucional da doença, no estado, foram extraídos do Cadastro Nacional de Estabelecimentos de Saúde (CNES),^b^ que disponibiliza informações acerca da rede assistencial nas três esferas do governo (municipal, estadual e federal). Dessa forma, foi possível o georreferenciamento dos serviços no mapa construído com as taxas de aids.

A unidade de análise foi o município. As variáveis estudadas foram as taxas médias de aids por município e a localização geográfica dos SAE. Utilizaram-se as coordenadas geográficas dos endereços das instituições e o código do Instituto Brasileiro de Geografia e Estatística (IBGE) referente ao município de residência das pessoas notificadas com a doença no período estudado.

O estado do Ceará localiza-se na região Nordeste do Brasil. Dividido em sete mesorregiões, compreende 184 municípios distribuídos em uma área de aproximadamente 148.825,6 km^2^. Sua população é estimada em aproximadamente 8,6 milhões de habitantes, em 2012, com densidade populacional de 57,8 hab./km^2^ (Figura 1).^a^

**Figura 1:** Mapa dos municípios e mesorregiões do estado do Ceará segundo censo demográfico de 2010. Brasil, 2014.

Foram avaliados 7.896 indivíduos diagnosticados com aids, na faixa etária de 13 anos ou mais, que residiam no Ceará, notificados entre os anos de 2001 a 2011 pela ficha de investigação do Sistema de Informação de Agravos de Notificação (Sinan).^c^ Foram excluídos casos duplicados e notificações repetidas.

A taxa média da aids por município foi calculada para os períodos de 2001 a 2006 e de 2007 a 2011. No primeiro período, o número médio de casos e a população média foram obtidos pela soma do número de casos ocorridos entre 2001 e 2006 e pela soma da população estimada de cada ano, ambos divididos por seis. No segundo período, a média dos casos e a média da população foram divididas por cinco. A taxa em cada período representa o número de casos médios dividido pela população média, multiplicado por 100.000 habitantes.

Realizou-se também a medida do índice de Moran global sobre a taxa média para testar a existência de autocorrelação espacial. Esse índice, cujo cálculo permite testar se áreas vizinhas diferem do que seria esperado em um padrão de completa aleatoriedade espacial, assume valores que variam de -1 a +1. A agregação espacial é expressa por valores positivos, enquanto os negativos expressam autocorrelação inversa, verificando-se ausência de autocorrelação espacial quando seu valor tende a zero.^16^

A presença de agregados espaciais foi avaliada pelo índice I de Moran local (*Local Indicators of Spatial Association* – LISA) e apresentados no LisaMap. Esse índice possibilita identificar a dependência espacial e quantificar o grau de associação espacial em cada localidade do conjunto amostral. Os valores de significância são divididos em quatro grupos: não significantes, com significância de 95,0%, com significância de 99,0% e com significância de 99,9%.^14^

O BoxMap foi utilizado para visualização gráfica do diagrama de espalhamento de Moran, que divide a região em quadrantes com características específicas. As áreas localizadas nos quadrantes Q1 (Alto-Alto) e Q2 (Baixo-Baixo) possuem autocorrelação espacial positiva, significando cidades com valores das taxas de aids semelhantes às suas vizinhas, caracterizando agregados espaciais. Os quadrantes Q3 (Alto-Baixo) e Q4 (Baixo-Alto) constituem autocorrelação espacial negativa, com cidades apresentando taxas da doença discrepantes entre as localidades próximas.^14^

Para descrever as áreas de influência dos SAE, construiu-se o Diagrama de Voronoi a partir dos pontos formados pelas coordenadas geográficas do endereço de cada instituição distribuídos nos dois períodos do estudo. Este diagrama permite definir regiões associadas a cada ponto individual em um mapa, em vez de segmentar uma área geográfica em limites legalmente definidos, cujos polígonos são divididos no espaço de acordo com pontos, eventos, ou ambos.^7^ Esta abordagem, útil em pesquisas epidemiológicas, permite detectar aglomerados de pontos em um determinado espaço-tempo.^13^

Foram construídos mapas para os períodos de 2001 a 2006 e 2007 a 2011 com as taxas de aids e a distribuição espacial dos SAE. Foi identificado se as ações de saúde disponíveis no estado se distribuem próximas de áreas com altas densidades de casos de aids. Utilizou-se o programa QuantumGis^®^ v.2.4.0.

Este estudo é parte da investigação ampliada sobre a distribuição espacial e determinantes sociais de saúde na população com aids no Ceará, aprovada pelo Comitê de Ética da Secretaria de Saúde do Estado do Ceará (Protocolo 203.911).

## RESULTADOS

A distribuição da aids nos dois períodos estudados apresentou aglomerações dos casos nas seguintes regiões: capital cearense e no seu entorno; Noroeste Cearense, próximo à região fronteira com Piauí; e norte do Jaguaribe (Figura 2 - Mapas 1A e 2A). Foram observadas baixas concentrações nos municípios e em seus respectivos vizinhos na região central e Sul Cearense.

**Figura 2:** Distribuição geográfica dos Serviços de Assistência Especializada (SAE), taxas médias (mapas 1A e 2A), BoxMap (mapas 1B e 2B) e LisaMap (mapas 1C e 2C) das taxas de aids segundo Diagrama de Espalhamento de Moran, no estado do Ceará, Brasil, 2001-2011.

Quase todo o litoral do estado apresentou altas taxas de aids (Figura 2 - Mapas 1A e 2A), e a relação das taxas de um município com sua vizinhança apresentou o mesmo padrão Alto-Alto (BoxMap - Figura 2 - Mapas 1B e 2B). Comparando o primeiro período com o segundo, nota-se a expansão da doença no oeste do estado, com maior quantidade de municípios no padrão Alto-Baixo ou Baixo-Alto. O LisaMap (Figura 2 - Mapas 1C e 2C), por sua vez, mostra que as taxas de aids e sua correlação espacial com as regiões vizinhas no período de 2001 a 2006 são estatisticamente significantes em Fortaleza (capital do estado) e parte da Região Metropolitana, em Sobral (região central do Noroeste Cearense) e no Sul Cearense. Após aplicação do método Lisa, observou-se significativo crescimento da aids nos municípios do sudoeste dos Sertões Cearenses, anteriormente não atingidos pela doença. Entre 2007 e 2011, a aids distribuiu-se significativamente em maior número de municípios que no período anterior, ainda nas mesmas regiões (Metropolitana, Noroeste Cearense e Sul Cearense).

Confrontando a distribuição dos SAE com a distribuição da aids no estado (Figura 2), observaram-se oito instituições inicialmente concentradas em Fortaleza apresentando *cluster* de aids estatisticamente significante. Entretanto, aglomerados da doença em outras localidades, como o oeste dos Sertões Cearenses e o sul do Jaguaribe, não possuíam SAE próximos. No segundo período, observou-se acréscimo de dois SAE: um deles instalado em Fortaleza e outro no norte do Jaguaribe, localidade com elevadas taxas da doença. Entretanto, existia apenas um SAE em região com significativa aglomeração de casos no Noroeste Cearense e não foi instalado outro serviço.

Aplicando o diagrama de Voronoi na distribuição dos SAE (Figura 3), observou-se que os serviços mais distantes da capital cearense abrangiam maior área geográfica. No período de 2001 a 2006 (Figura 3 - Mapa 1A), os polígonos relacionados aos SAE não conseguiram influenciar toda a extensão do estado, indicando possíveis localizações com grande distância do serviço. O período de 2007 a 2011 (Figura 3 - Mapa 1A) possuía polígonos em quase todo o Ceará. Ademais, existia um polígono sem nenhum SAE, localizado na porção oeste do estado.

**Figura 3:** Distribuição de Serviço de Assistência Especializada (SAE) e polígonos de Voronoi. Ceará, 2001-2011.

## DISCUSSÃO

Apesar da concentração da aids na Região Metropolitana, o estado do Ceará apresenta aglomerações da doença em outras regiões, como o norte do Jaguaribe, Noroeste Cearense e oeste dos Sertões Cearenses, presentes ao longo dos dois períodos do estudo. No contexto da interiorização da epidemia, o acesso aos serviços de saúde tem implicância sobre a qualidade de vida das pessoas vivendo com HIV/aids.^5^ Ao contrário, a centralização dos serviços de assistência especializada se confronta com a expansão geográfica da epidemia, dificultando o acesso da população que mora mais distante, sendo menos assistida e favorecida.^11^

Verificou-se também a predominância dos SAE e dos *clusters* de aids na Região Metropolitana, o que pode influenciar na acessibilidade dos pacientes a esses serviços. Existe o consenso de que a concentração de atendimento em unidades de referência pode dificultar a acessibilidade geográfica do paciente, pois há mais dispêndio com transporte, licença no trabalho, entre outros fatores. Entretanto, pessoas que apresentam doenças estigmatizantes, como no caso do HIV/aids, preferem que seus domicílios não sejam tão próximos às unidades de assistência, na perspectiva da manutenção do anonimato e do não reconhecimento por outros indivíduos que frequentam o serviço.^1^

A pesquisa indica desproporção da quantidade de SAE no noroeste e no oeste dos Sertões Cearenses em relação às taxas de aids, os quais possuem elevados valores. Entretanto, um SAE foi criado no Jaguaribe no segundo período, acompanhando a demanda da localidade em relação à assistência às pessoas vivendo com HIV/aids.

A distribuição espacial da localização de unidades de saúde interfere diretamente na acessibilidade geográfica que, no caso de grandes distâncias aliada a tempo de marcha e ausência de transporte, compromete o tratamento de pessoas que necessitam de assistência especializada.^6^

Ainda, a decisão, pelos gestores, de implantar um serviço de saúde deve surgir a partir das demandas. Essas podem ser monitoradas por diversos sistemas de informação em saúde e junto a pesquisadores, instituições acadêmicas e usuários do Sistema Único de Saúde,^19^ subsidiando planejamento e prospecção logística dos serviços implantados. No Brasil, esses sistemas são pouco utilizados em municípios de menor porte, o que pode ocasionar a implantação de serviços de saúde em locais com pouca demanda, em detrimento de regiões que necessitem de mais unidades de saúde.^9^

A região centro-sul cearense não apresentou nenhum SAE. A distância dos municípios menores para os grandes centros urbanos, que concentram os SAE, dificultam o acesso aos recursos disponibilizados por esses serviços. Isso torna as pessoas mais vulneráveis às consequências da doença, o que constitui um desafio para os avanços contra essa epidemia.^1^

O estudo limita-se pela ausência de pesquisas semelhantes, dificultando o confrontamento dos dados obtidos com a situação em outras localidades. Não foram possíveis informações sobre o número mínimo de SAE preconizados por população ou pessoas vivendo com HIV/aids, dificultando análise de maior amplitude sobre a disponibilidade do serviço no estado.

Sugere-se a necessidade de estudos com abordagem qualitativa na compreensão da dinâmica social e comportamental da relação indivíduo/SAE, o que poderia ampliar o entendimento dessas distâncias ou proximidades com os serviços de apoio.

A informação sobre a epidemia da aids ainda necessita de avanços para viabilizar o geoprocessamento e a análise espacial. Além disso, é necessário que haja retorno das informações geradas por meio das pesquisas às instituições de saúde e políticas para a mudança da realidade apreendida pelos estudos.^17^

O estudo mostrou que o interior do estado possui tanto localizações com altas taxas de aids e baixa quantidade de SAE, quanto o inverso, apontando regiões que podem ser favorecidas com a implantação de novos serviços, como algumas localidades que foram beneficiadas no segundo período do estudo. A atenção básica pode contribuir na assistência a essa clientela, visto que possibilita maior acessibilidade, reservando as instituições secundárias e terciárias para situações mais complexas.

O recurso metodológico pode ser ampliado, na perspectiva de que os SAE interferem no processo saúde-doença de pessoas vivendo com HIV/aids, pois auxiliam nas atividades de assistência, prevenção e tratamento desses pacientes.

## Footnotes

a Instituto de Pesquisa e Estratégia Econômica do Ceará. Ceará em números. Fortaleza: Instituto de Pesquisa e Estratégia Econômica do Ceará; 2014 [citado 2014 mai 21]. Disponível em: http://www.ipece.ce.gov.br/

b Ministério da Saúde. Cadastro Nacional de Estabelecimentos de Saúde. Brasil: Cadastro Nacional de Estabelecimentos de Saúde; 2015 [citado 2015 mai 2]. Disponível em: http:// http://cnes.datasus.gov.br/

c Datasus. Ministério da Saúde. Sistema de Informação de Agravos de Notificação. Brasil: Sistema de Informação de Agravos de Notificação; 2012 [citado 2012 mai 2]. Disponível em: http://www2.datasus.gov.br/

## DESTAQUES

A análise da distribuição de serviços especializados e sua relação com a distribuição espacial da doença podem auxiliar no planejamento dos programas de assistência e controle. Esta análise constatou a concentração de Serviços de Assistência Especializada a portadores de HIV/aids na Região Metropolitana do estado do Ceará, onde há maior aglomeração de casos de aids. Tanto o Noroeste cearense como o Oeste dos Sertões Cearenses têm elevadas taxas de aids, com menor número de serviços especializados. No período posterior, dois SAE foram implantados no Jaguaribe, onde existiam aglomerados de casos da doença. O uso do Diagrama de Voronoi como possibilidade de identificar a cobertura territorial dos SAE mostrou serviços localizados fora da Região Metropolitana com grande extensão territorial, em detrimento de regiões sem nenhum serviço.

Os resultados podem ser incorporados no planejamento de distribuição dos serviços de saúde e na construção das redes assistenciais. Além disso, o método utilizado poderá ser replicado nos diversos espaços de saúde, identificando áreas prioritárias para a implantação de serviços.

Rita de Cássia Barradas Barata

Editora Científica
